# Biodegradable Microcapsules Loaded with Nerve Growth Factor Enable Neurite Guidance and Synapse Formation

**DOI:** 10.3390/pharmaceutics13010025

**Published:** 2020-12-25

**Authors:** Olga Kopach, Anton M. Pavlov, Olga A. Sindeeva, Gleb B. Sukhorukov, Dmitri A. Rusakov

**Affiliations:** 1 Department of Clinical and Experimental Epilepsy, UCL Queen Square Institute of Neurology, University College London, London WC1N 3BG, UK; 2 School of Engineering and Materials Science, Queen Mary University of London, Mile End Road, London E1 4NS, UK; a.pavlov@qmul.ac.uk (A.M.P.); O.Sindeeva@skoltech.ru (O.A.S.); 3 Remote Controlled Theranostic Systems Laboratory, Saratov State University, 83 Astrakhanskaya Street, 410012 Saratov, Russia; 4 Center for Neurobiology and Brain Restoration, Skolkovo Institute of Science and Technology, 3 Nobel Street, 143005 Moscow, Russia

**Keywords:** layer-by-layer (LbL) microcapsules, nerve growth factor (NGF), axon guidance

## Abstract

Neurological disorders and traumas often involve loss of specific neuronal connections, which would require intervention with high spatial precision. We have previously demonstrated the biocompatibility and therapeutic potential of the layer-by-layer (LbL)-fabricated microcapsules aimed at the localized delivery of specific channel blockers to peripheral nerves. Here, we explore the potential of LbL-microcapsules to enable site-specific, directional action of neurotrophins to stimulate neuronal morphogenesis and synaptic circuit formation. We find that nanoengineered biodegradable microcapsules loaded with nerve growth factor (NGF) can guide the morphological development of hippocampal neurons in vitro. The presence of NGF-loaded microcapsules or their clusters increases the neurite outgrowth rate while boosting neurite branching. Microcapsule clusters appear to guide the trajectory of developing individual axons leading to the formation of functional synapses. Our observations highlight the potential of NGF-loaded, biodegradable LbL-microcapsules to help guide axonal development and possibly circuit regeneration in neuropathology.

## 1. Introduction

The depletion of endogenous growth factors in the adult brain has been associated with numerous neurodegenerative conditions that can lead to severe neurological and psychiatric disorders [1,2,3]. Among at least 50 identified growth factors in the nervous system, several are considered essential for survival, maintenance, and regeneration of nerve cells. These include nerve growth factor (NGF), brain-derived neurotrophic factor (BDNF), and glial cell-derived neurotrophic factor (GDNF). Supplying neurotrophic agents to specific neuronal subpopulations that undergo degeneration produced therapeutic effects, as documented by clinical trials dealing with Alzheimer’s and Parkinson’s diseases [4,5]. Several strategies have been attempted to help deliver NGF into the brain [6], aiming to overcome the undesired concomitants of systemic application, such as limited blood–brain barrier penetration or severe side effects. The viral vector-mediated gene therapy could potentially target identified neuronal populations but once delivered it could not be easily terminated, thus raising major safety issues. As an alternative route, cell-based approaches have been tried, such as transplanting NGF-secreting induced pluripotent stem cell (iPSC)-derived neurons into the damaged brain [7,8]. Although this method generates sustained endogenous production of NGF for up to a few months, it consistently showed a low cell survival rate following transplantation. An elegant innovative approach combining pharmaceutical and material sciences has been attempted, by encapsulating the NGF-secreting iPSC-derived cells, aiming to protect them from the immune response following transplantation [9,10,11,12]. Although the safety of this platform was confirmed, the NGF secretion level remained too low in half of the cohort [13].

An alternative strategy sought the encapsulation of exogenous NGF, to enable its prolonged release within the targeted area. Numerous studies have been carried out exploring different types of the cargo system, including biodegradable poly-lactic-co-glycolic-acid (PLGA) microspheres [14,15,16,17] and nanoparticles made from natural polymer chitosan [18,19], gelatin [20,21], or collagen [22]. Most of these methods have focused on the peripheral nervous system or immortalized cell lines in vitro. The efficiency of the encapsulation technology and its applications with respect to central neurons has thus remained poorly understood. Here, we sought to test the effects of encapsulated NGF on the morphogenesis of central neurons, by exploring microcapsules that are nanoengineered from biodegradable materials, poly-L-arginine and dextran [23,24,25] using the layer-by-layer (LbL) technique [26,27]. We have previously demonstrated biocompatibility, and key properties of release and biodegradation for these LbL-fabricated capsules, both in acute tissue slices and up to two weeks in free-behaving rodents, with the potential therapeutic efficacy as a local source of anaesthetics, and no detectable adverse effects [24]. This technique enables control over the capsule size and allows for encapsulation of various cargos, of a medium to high molecular weight. It also provides the possibility to set key properties of cargo storage and release. The established properties of LbL-microcapsules make this system potentially well-suited for the delivery of NGF to selected populations of brain neurons. To understand its potential, we, therefore, encapsulated NGF within the core of LbL-microcapsules and explored the effect of microcapsules on the morphogenesis of hippocampal neurons in vitro.

## 2. Materials and Methods

### 2.1. Fabrication of Microcapsules Carrying NGF

Polyelectrolyte-based microcapsules were fabricated using the LbL-assembly technique [28,29]. Briefly, CaCO_3_ microspheres with co-precipitated neurotrophin NGF were used as sacrificial templates. To this end, 1 mL of 0.33 M Na_2_CO_3_ was added to 1 mL of 0.33 M CaCl_2_ with 40 µg NGF supplemented with 200 µg bovine serum albumin (BSA) while stirring vigorously. BSA is widely used as an inert filler, for co-encapsulation with small quantities of biologically active substances, particularly with growth factors [30]: it acts as a protective agent and protein stabilizer [31]. As shown earlier for fibroblast grow factor in polyelectrolyte microcapsules, when co-encapsulated with BSA growth factors display more efficient immobilization and preservation of biological activity [32]. After the successful synthesis of the cores (verified by optical microscopy), the templates were washed with ddH_2_O (by 3 steps of centrifugation/resuspension), and layers were assembled using the procedure as we have previously described in detail [24]. Shells were assembled of 3 bilayers of positively charged poly-L-arginine (PArg) and oppositely charged dextran sulfate sodium salt (DS), with the resulting shell structure [PArg/DS]_3_. Both polyelectrolytes were adsorbed from 2 mg/mL solutions in 0.15 M NaCl. At the final step, the CaCO_3_ cores were dissolved in 0.2 M EDTA (pH 6.5 with NaOH).

### 2.2. Materials

For the microcapsule fabrication purposes, all polyelectrolytes and other chemicals, including PArg, DS, BSA, NGF-β (molecular weight 13.5 kDa), and all salts were purchased from Sigma-Aldrich (Gillingham, UK).

A hemocytometer for counting the number of microcapsules and freshly isolated hippocampal neurons was purchased from Labtech International (Heathfield, UK). For determining protein amount, Bradford assay kit was purchased from Sigma-Aldrich (Gillingham, UK). NeuroBasal A medium and B27 supplement were purchased from ThermoFisher Scientific (Hemel Hempstead, UK). The FM1-43 dye (*N*-(3-Triethylammoniumpropyl)-4-(4-(Dibutylamino)Styryl) Pyridinium Dibromide) and TRITC (tetramethylrhodamine) were also purchased from ThermoFisher Scientific (Hemel Hempstead, UK).

### 2.3. Encapsulation Efficiency

The amount of NGF per capsule was estimated based on the amount of co-loaded BSA in a similar encapsulation protocol, as described in detail previously [23,33]. BSA encapsulation ratio was ~67%, as we have determined with the Bradford protein assay using a Lambda 950 UV/VIS spectrophotometer (PerkinElmer, Beaconsfield, UK). Accordingly, the amount of NGF encapsulated per capsule could be estimated as ~0.27 pg, which, upon release, should fall within a wide range of functionally relevant NGF concentrations [34,35].

### 2.4. Scanning Electron Microscopy (SEM)

**Figure 1:**
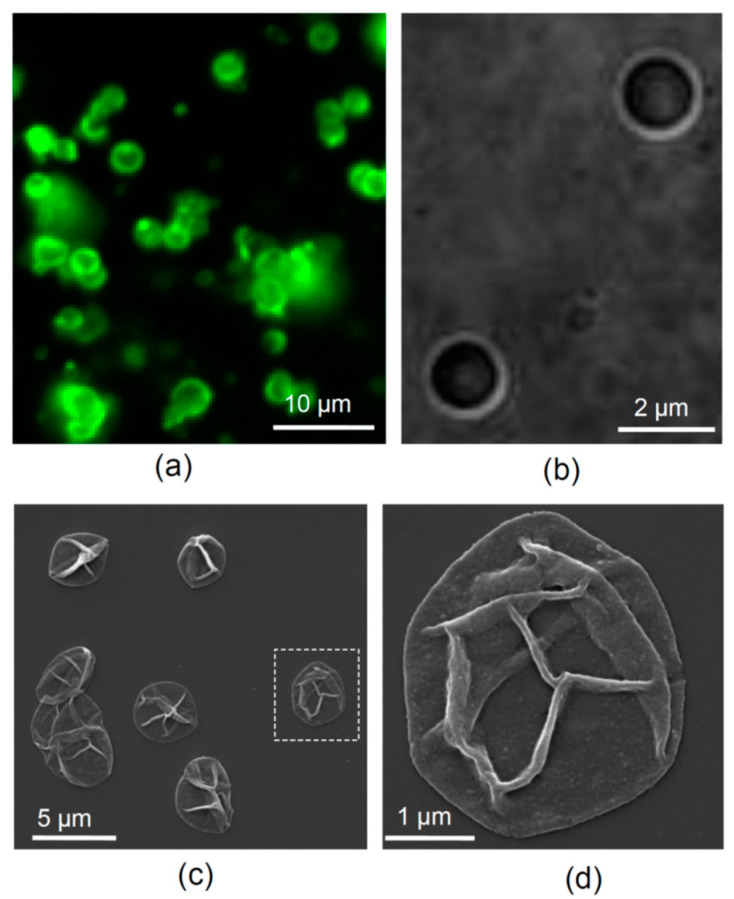
LbL-microcapsules carrying NGF. (**a**) An example of LbL-microcapsules in a suspension (epifluorescence of FITC conjugated to the microcapsule shell). (**b**) A DIC image of microcapsules at higher magnification. (**c**) SEM images of LbL-microcapsules dried and sputtered with gold. (**d**) Dotted rectangle regions in (**c**) shown at higher magnification.

To visualize fabricated microcapsules, we used scanning electron microscopy (SEM; FEI, Inspect-F, Hillsboro, OR, US) to ensure the structural appropriateness of our samples. To this end, a droplet of dispersing suspension of microcapsules was plated on a glass slide attached to the sample holder, then dried and sputtered with gold (Figure 1c,d). SEM was carried out at accelerating voltage of 10 kV and a working distance of 10 mm.

### 2.5. Primary Nerve Cell Cultures

Hippocampal neurons were isolated from the Sprague-Dawley rat pups (P0 to P2 day-old) in accordance with the European Commission Directive (86/609/EEC) and the United Kingdom Home Office (Scientific Procedures) Act (1986), using animal protocols adapted in-house [36,37]. Neurons were cultured in a NeuroBasal A/B27-based medium on a rat astrocyte feeder layer at 37 °C in a humidified atmosphere containing 95% O_2_ and 5% CO_2_ as we have described in our previous work [24]. We purposely maintained the low-density cultures to foster growth of developing hippocampal neurons with a polarized morphology [38]. For the low-density cultures, hippocampal neurons were plated at a low density, ~20,000 cells per coverslip. Neuronal morphology was assessed for individual cells at earlier developmental stages (from a few hours after plating to up to one week).

### 2.6. Introducing Encapsulated NGF to Neuronal Cultures

Microcapsules carrying NGF were added to the cultures shortly after plating cells on glass coverslips, at 1.5–2 h post-plating. To this end, an aliquot of freshly made microcapsules, re-suspended in a culture medium, was gently added at a total volume of 50 µL into the well containing plated neurons. This amounted to ~2.5 × 10^6^ microcapsules per well; accordingly, the amount of encapsulated NGF added could be estimated as 670–680 ng/mL. Neuronal cultures with NGF were incubated at 37 °C (95% O_2_/5% CO_2_) and cell morphology was systematically examined at 6.5 h, 24 h, and 7 d of incubation with encapsulated NGF.

### 2.7. Assessment of Neuronal Morphology and the Neurite Outgrowth Rate

Neuronal morphology was assessed at different time-points after incubation with encapsulated NGF. For live imaging, a coverslip with cultured cells was placed in a recording chamber mounted on the stage of an Olympus BX51WI upright microscope (Olympus, Japan) equipped with a LUMPlanFI/IR 40 × 0.8 objective, tuned for infrared differential interference contrast (DIC) imaging. Cells were continuously superfused with a Ringer solution, a physiological buffer containing (in mM) 126 NaCl, 3 KCl, 2 MgSO_4_, 2 CaCl_2_, 26 NaHCO_3_, 1.25 NaH_2_PO_4_, 10 D-glucose, continuously bubbled with 95% O_2_ and 5% CO_2_ (pH 7.4; 300–310 mOsmol). Imaging was carried out using a Photometrics Evolve 512 EMCCD camera (Cairn Research, Faversham, UK) at a frame mode of 512 × 512 pixels, using various digital zooms. A Lumen Dynamics X-Cite 120Q lamp (Feasterville, PA, USA) was used as a light source to visualize microcapsules by TRITC (or FITC) fluorescence conjugated to their shell (Figure 1a). Images of individual hippocampal neurons (100–150 nm/pixel) were acquired using Micromanager 4.1 software (NIH, Bethesda, MD USA).

To quantitatively compare morphological parameters among experimental groups, the lengths and branching order of neurites were documented at the corresponding time-points in control (untreated) cultures and those supplemented with encapsulated NGF. Neurites were traced manually, at varied magnification, categorized off-line for the primary neurites, secondary-order, and tertiary branches, and quantified using a NeuronJ plugin of ImageJ software (NIH, Bethesda, MD, USA).

### 2.8. Visualizing Functional Synapses with FM1-43 Dye

To confirm axonal development and formation of functional neural connections, we visualized functional synapses using a well-rehearsed method based on the fluorescent lipophilic dye FM1-43, in line with the protocol established by us earlier [39]. FM1-43 binds to cell membranes, and during active neurotransmission is taken up during the endocytosis phase of vesicle recycling, thus filling presynaptic terminals with fluorescing vesicles [40]. To enable this protocol, we added 20 µM FM1-43 dye to cultured neurons and depolarized hippocampal neurons using bath application of 50 mM KCl in a HEPES-based physiological buffer for 1–2 min, thus triggering massive synaptic activity. This was followed by washing out all externally exposed FM1-43. Experiments were performed in a HEPES-based physiological buffer containing (in mM) 135 NaCl, 5 KCl, 2 CaCl_2_, 2 MgCl_2_, 10 HEPES, 10 glucose (pH 7.4). Images were acquired using Micromanager 4.1 software (NIH, Bethesda, MD, USA) and analyzed off-line, using a freely available ImageJ software (NIH, Bethesda, MD, USA).

### 2.9. Statistics

We routinely presented the data as mean ± standard error of the mean (s.e.m.), with n referring to the number of cells analyzed within experimental groups. As the sample size was consistently above 20, the normality test had no practical purpose as the Central Limit Theorem would predict the Gaussian distribution of the mean. Because we considered parameter variability either among individual cells or among individual neurites, with no biological hypotheses pertaining to the multi-factorial effects, we formed and compared the samples accordingly. The Student’s *t*-test (two-tailed unpaired) was used to determine the statistical difference between such groups. Testing was carried out using OriginPro (OriginLab, Northampton, MA, US) and Excel software packages. The null hypothesis rejection level at *p* < 0.05 was considered statistically significant.

## 3. Results

### 3.1. Microcapsules

To visualize microcapsules in the light microscope, the fluorescently labelled TRITC- or FITC-conjugated polymer (commercially available FITC-labelled poly-L-lysine was used instead of PArg in the latter case) was utilized for the third-layer fabrication (Figure 1a,b).

Microcapsules had an average diameter of 2–3 µm (Figure 1a,b), and the nanoscopic appearance of their thin shells could be revealed by scanning electron microscopy (SEM; Figure 1c,d). The number of microcapsules in a freshly prepared suspension was ~50 × 10^6^ mL^−1^, as determined using a haemocytometer, with a total sample volume of 2 mL. The suspension of microcapsules in ddH_2_O was stored at 4 °C and re-suspended immediately before supplementing to neuronal cultures.

### 3.2. Encapsulated NGF Boosts the Neurite Outgrowth Rate

To explore the effects of encapsulated NGF, we employed low-density neuron-astrocyte co-cultures, which provide favourable physiological conditions for synapse formation and function. In addition, in low-density neuronal cultures, hippocampal neurons develop with clearly polarized morphology [38], which facilitates quantitative assessment of neuronal development across its distinct stages. Cultured hippocampal neurons were assessed individually for their morphology, at several stages of development, either in the presence of LbL-microcapsules loaded with NGF or in the age-matched control (untreated) cultures.

**Figure 2:**
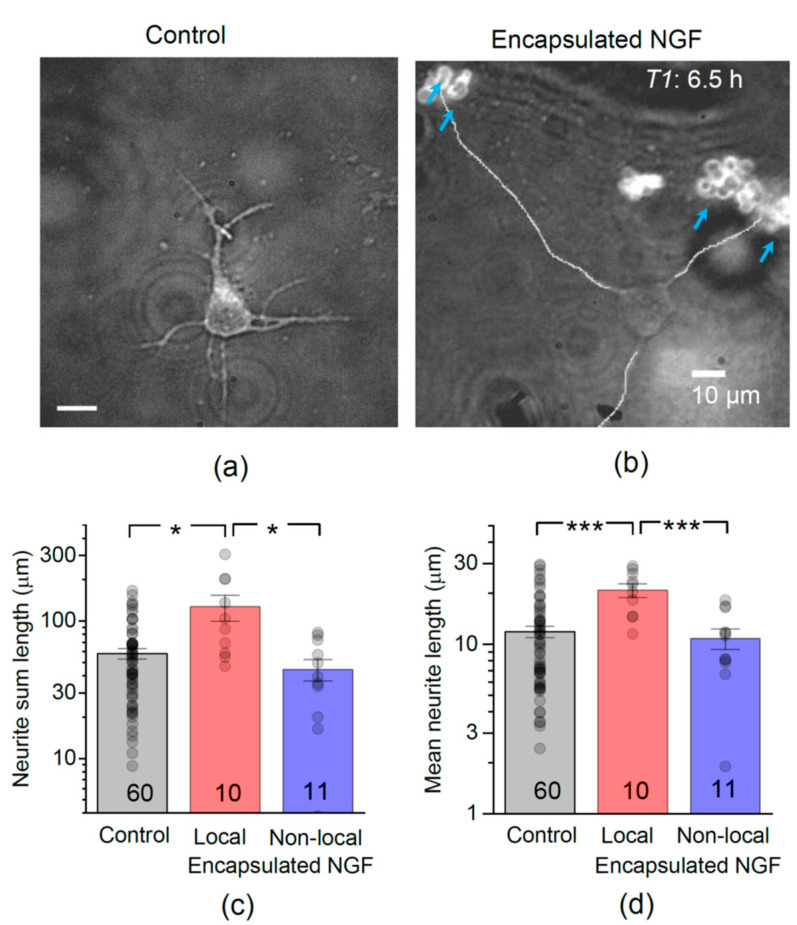
Encapsulated NGF boosts neurite outgrowth in developing hippocampal neurons in vitro. (**a**) A snapshot of developing hippocampal neurons, at 6.5 h after plating, in control conditions. (**b**) As in (**a**) but in the presence of NGF-loaded LbL-microcapsules; blue arrows, clusters of microcapsules labelled by TRITC; merged DIC and TRITC fluorescence channels; line-traced neurites illustrate neurite length measurement in individual cells. (**c**) Statistical summary of total neurite length (sum of individual neurite lengths) per neuron, 6.5 h after plating, in control conditions (no microcapsules), and in the presence of encapsulated NGF for local (<50 µm from capsules) and non-local (>50 µm away) cells, as indicated; numbers of cells shown. Dots, individual cell data; bars, mean ± s.e.m; * *p* < 0.05 (two-tailed unpaired *t*-test); ordinate, log scale. (**d**) Statistical summary for the mean neurite length per individual cell; *** *p* < 0.001 (two-tailed unpaired t-test); other notations as in (**c**).

In control co-cultures, nerve cells displayed a prompt morphological development, which was consistent with the established developmental stages in culture [38,41]. The vast majority of neurons had several clearly distinguishable small processes as early as 6.5 h after plating (developmental stages 1 and 2, in accord with [38]). Within the first 24 h, all examined neurons exhibited well-formed polarity and numerous extended neurites (developmental stages 2 and 3; see below). We next measured (i) the total accumulated length of all neurites (dendrites and axons), and (ii) the mean neurite length per cell, in control cultures (Figure 2a) and in those containing NGF-loaded LbL-microcapsules (Figure 2b).

We found that encapsulated NGF markedly boosted the development of hippocampal neurons. At 6.5 h after plating, the total length of neurites was 58 ± 4.9 µm in control (mean ± s.e.m., here and thereafter; n = 60 neurons, 331 neurites), whereas in the presence of NGF-loaded microcapsules it was 82 ± 14.6 µm (n = 21 neurons, 104 neurites), an increase of ~41%. In most cases, the outgrowing neurites were directed towards the clusters of microcapsules, rather than into an arbitrary direction (Figure 2b). The role of glia in mediating NGF action in cultures was previously ruled out [42], thus pointing to the encapsulated NGF as a direct effector. The boosting effect of encapsulated NGF, as opposed to the presence of capsules in the medium, was confirmed by its local nature: only neurons growing within ~50 µm of the NGF-loaded microcapsules (termed ‘local neurons’ thereafter) exhibited elongated neurites. The total length of neurites almost doubled in these local neurons (n = 59 neurites in 10 neurons; *p* < 0.05 compared to control; Figure 2c). There was no significant difference in the total neurite length between neurons located >50 µm from microcapsules (termed ‘non-local neurons’ thereafter; 44 ± 7.9 µm, n = 45 neurites in 11 neurons) and those in control cultures (*p* < 0.05; Figure 2c). Correspondingly, the mean neurite length was also increased in test cultures: at 6.5 h post-plating, it was 16 ± 1.7 µm (n = 21 cells, *p* < 0.05) compared to 12 ± 0.9 µm (n = 60 cells) in control cultures (Figure 2d). Again, only local neurons (n = 10) exhibited the increase (*p* < 0.001 compared to control; Figure 2d).

**Figure 3:**
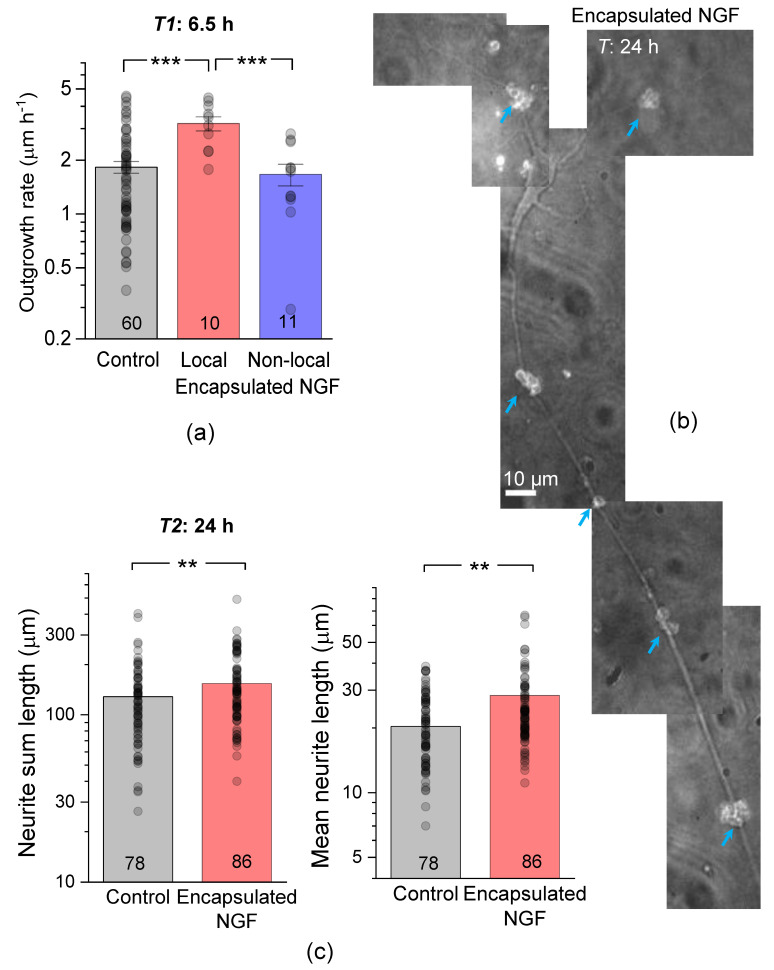
Encapsulated NGF elevates the neurite growth rate. (**a**) Average neurite length and the estimated outgrowth rate in control conditions and in the presence of encapsulated NGF for local (<50 µm away from capsules) and non-local (>50 µm away) cells, as indicated. (**b**) A snapshot of a pyramidal-like hippocampal neuron at 24 h, with the growth trajectory of its main neurites directed by the clusters of NGF-releasing capsules (blue arrows; TRITC microcapsule labelling). Image, merged DIC and TRITC fluorescence channels (grey-level). (**c**) Statistical summary of the total (sum) neurite length (left) and mean neurite length (right) per individual neuron, at 24 h post-plating in control cultures and in the presence of encapsulated NGF. Dots, individual cell data; bars, mean ± s.e.m; numbers of analysed cells shown; ** *p* < 0.01, *** *p* < 0.005 (two-tailed unpaired *t*-test); ordinate, log scale.

At this development stage, the neurite outgrowth rate was ~1.8 µm h^−1^ in control neurons, but ~3.2 µm h^−1^ in local neurons, an increase of ~78% (Figure 3a). The neurite outgrowth rate remained unchanged (~1.7 µm h^−1^) in non-local neurons (Figure 3a).

At 24 h after plating, cell neurites displayed a clear trajectory preference towards NGF-loaded capsules, which appeared to guide and accelerate their growth (Figure 3b). The total length of neurites in our control co-cultures was 125 ± 7 µm (n = 489 neurites in 78 neurons; Figure 3c), thus displaying a clear developmental advantage over neuronal mono-cultures explored earlier (developmental stages 2 and 3 [38]). In the presence of encapsulated NGF, neurite outgrowth was boosted further still: the total neurite length increased by ~26% (n = 555 neurites in 86 neurons, *p* < 0.01 compared to the age-matched control; Figure 3c). The mean neurite length was also increased, by a similar degree (a ~21% increase, *p* < 0.01 compared to control; Figure 3c). Such prompt elongation led to almost all neurites eventually appearing within 50 µm to microcapsule clusters, making further separation of ‘local’ and ‘non-local’ neurons infeasible.

After >24 h of incubation with NGF-loaded microcapsules, hippocampal neurons could be morphologically classified as ones reaching the developmental stages 4 and 5 (‘dendritic outgrowth’ and ‘maturation’ [38]). At this stage, the growing neurites become overlapped and intermingled, making the identification of individual neurites no longer feasible for the present analyses.

### 3.3. Encapsulated NGF Enhances Neurite Branching

**Figure 4:**
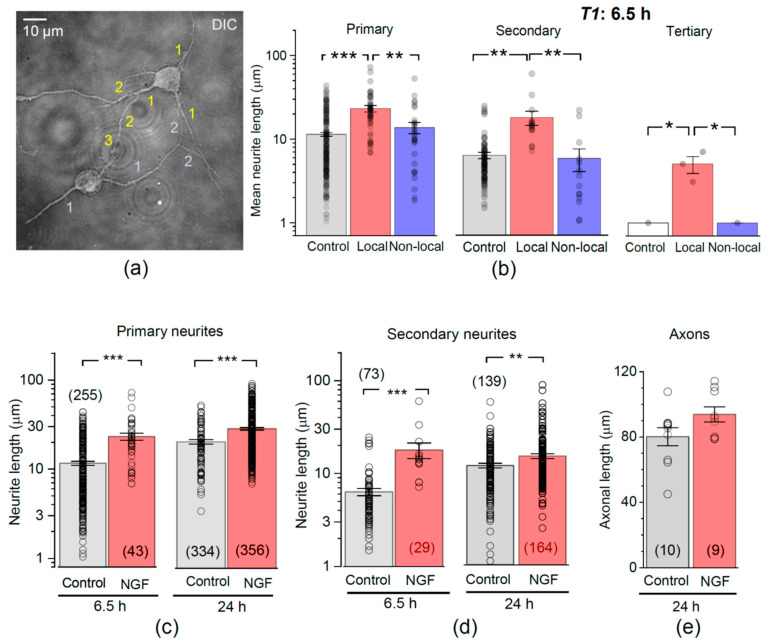
Encapsulated NGF boosts neurite branching. (**a**) Example (DIC channel) of two hippocampal neurons at 6.5 h post-plating in neuron-astrocyte co-culture, with the primary, secondary, and tertiary neurites as indicated (1, 2, and 3, respectively). (**b**) Statistics for individual neurite lengths, at various branch order, in control and in the presence of encapsulated NGF, for local (<50 µm from capsules) and non-local (>50 µm) neurons, as indicated. Dots, neurite measurements pooled for individual cells; bars, mean ± s.e.m.; * *p* < 0.05; ** *p* < 0.01, *** *p* < 0.001 (two-tailed *t*-test). (**c**,**d**) Statistical summary for the length of primary (**c**) and secondary branches (**d**) at 6.5 h and 24 h post-plating in control and in the presence of encapsulated NGF, as indicated. Dots, individual neurite measurements; bars, mean ± s.e.m.; ** *p* < 0.01, *** *p* < 0.001 (two-tailed t-test). (**e**) Statistics for the length of individual axons, as identified at 24 h post-plating, in control and in the presence of NGF-loaded microcapsules, as indicated.

We next examined whether encapsulated NGF could influence branching of elongated neurites, a key element in the formation of axons and dendrites. Individual hippocampal neurons were examined for the length of primary neurites, followed by the secondary and tertiary branches, if any (Figure 4a). At 6.5 h post-plating, cells had predominantly primary neurites, with few secondary-order branches and a rare occurrence of tertiary branches. Overall, the mean length was ~11.4 µm for the primary neurites (n = 255) and ~6.3 µm for the secondary-order branches (n = 73; Figure 4b). In the presence of encapsulated NGF, hippocampal neurons developed, over the same period (6.5 h), both primary and secondary-order branches (both to a greater length than control), and even a few tertiary branches. Again, these acceleration effects were seen only in local neurons (<50 µm from microcapsules). The mean length of primary neurites was ~23.2 µm in local neurons compared to ~11.4 µm in control neurons (*p* < 0.001), and ~13.7 µm in non-local (>50 µm away from microcapsules) neurons (*p* < 0.01 compared to local neurons in the same culture). Correspondingly, the secondary-order neurite mean length was ~17.9 µm in local neurons compared to ~6.3 µm in control cultures (*p* < 0.01), and to ~5.8 µm in non-local neurons in the same culture (Figure 4b).

After 24 h of incubation with encapsulated NGF, the mean neurite length increased for both primary (by ~22%, *p* < 0.001) and the secondary-order branches (by ~23%, *p* < 0.01), as compared to the age-matched neurons in control (Figure 4c,d). At that time point, we could morphologically distinguish the main axonal branches, as opposed to dendrites, in accord with morphological criteria established by us earlier [43]. The mean axonal length was 80 ± 5 µm (n = 10 neurons) in control but appeared to increase to 94 ± 5 µm in the presence of encapsulated NGF (n = 9 cells, *p* = 0.07; Figure 4e).

### 3.4. Neurons Cultured with Encapsulated NGF Form Functional Synapses

**Figure 5:**
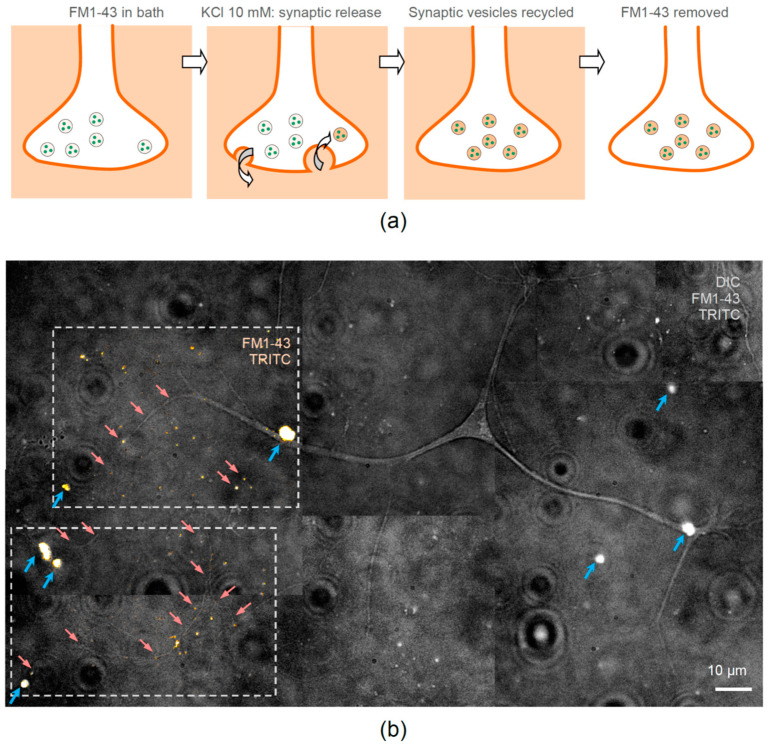
Neurites of hippocampal neurons are guided towards encapsulated NGF and form functional synapses. (**a**) A diagram sequence explaining fluorescence imaging of functional synaptic connections in cultured neurons using the fluorescent styryl dye FM1-43. The dye is washed in the bath, the active release of synaptic vesicles is activated by 50 mM KCl (bath application for 1 min), the recycled vesicles take up and store the dye inside the axonal terminal, and the external FM1-43 fraction is washed out leaving detectable fluorescence inside active synapses. (**b**) One neuron example in neuron-astrocyte co-culture, with neuronal main and smaller processes growing towards clusters of NGF-releasing LbL-microcapsules at 7 days in vitro. Grey-scale image, merged DIC, TRITC, and FM1-43 fluorescence; red arrows, examples of neurites growing towards clusters of LbL-microcapsules (blue arrows, TRITC labelling); dotted frames, two ROIs illustrating fluorescence of TRITC and FM1-43 fused with DIC image to highlight the FM1-43 labelled functional synapses (orange spots) revealed after KCl application.

To test whether the axonal development of hippocampal neurons in the presence of encapsulated NGF leads to functional synaptic connections between neurons, we carried out imaging of functional synapses in live hippocampal neurons using the fluorescent styryl dye FM1-43, the approach we validated earlier [39] (Figure 5a).

In brief, we bath applied FM1-43 and depolarized cultured neurons with high-potassium (50 mM KCl) for 1–3 min, after which the dye was washed out. The fluorescence signal that was accumulated by recycled synaptic vesicles inside presynaptic terminals during intense synaptic activity remained clearly visible (Figure 5a). Equipped with this approach, we were able to visualize the FM1-43-mediated fluorescence related to numerous bouton-like structures, either on neuronal soma or along neurites, in hippocampal neurons of 1-week-old cultures (Figure 5b). This observation confirms the formation of functional (active) synapses in hippocampal neurons at the age of 7 days in vitro in the presence of NGF-loaded microcapsules.

## 4. Discussion

The polyelectrolyte microcapsules fabricated by the LbL-technique have been demonstrated as an efficient delivery system for targeting different tissue and cell types. The suitability for this type of carriers has been confirmed in animals in vivo through either systemic application [44,45,46,47] or during local injections of the microcapsule suspension into the targeted tissue [24]. However, there have been only a few attempts to explore the use of biodegradable LbL-microcapsules in the central nervous system, especially with respect to the brain where multiple and distinct cell types are densely inter-connected with high spatial specificity. Therefore, our aim was to examine the potential of LbL-microcapsules for localised delivery of neurotrophins to central neurons, a potential tool to enhance guidance and formation of functional neuronal connections. This context differs from that of systemic application which envisages blood-brain barrier permeability followed by cell targeting.

Our strategy took advantage of the previous work in which fabrication protocols, release properties, and biodegradation in situ and in vivo for the LbL-fabricated microcapsules have been established [12,23,24,33]. Thus, the functional data obtained here in neuronal-astrocyte co-cultures, together with the previous reports pertaining to the encapsulation per se, suggest that LbL microcapsules meet the key requirements for applications targeting brain neurons. These include (i) high biocompatibility (no excitotoxicity) for all constitutive components of the microcapsule, (ii) gradual, prolonged release of the encapsulated compound of interest (including low molecular weight substances), and (iii) biodegradability (safe use). Monitoring primary hippocampal neurons in the present work has revealed no signs of excitotoxicity in the presence of microcapsules, at any stage of neuronal development and morphogenesis in vitro. Again, this is consistent with no effects of empty LbL-microcapsules (of the same composition) on primary hippocampal neurons or on peripheral nerve cells in vivo [24]. In addition, numerous studies have reported no toxicity produced by the LbL-microcapsules in other cell types of non-neuronal origin [25,33,48,49,50].

The LbL technique of microcapsule fabrication enabled the encapsulation of NGF (a ~13 kDa neuropeptide) using calcium carbonate particles as templates (for increasing the payload capacity, as validated previously [23,33]). NGF belongs to the superfamily of neurotrophins and has a diverse array of functions. These well-established properties include trophic effects and regulation of morphogenesis, such as guidance of nerve cell growth and axonal pathfinding, regulation of continued neurogenesis in the adult brain, and the activity-dependent network remodelling through establishing new synapses for synaptic transmission, plasticity, and neurocognitive functions [51,52]. This array of NGF actions involves regulated secretion by small amounts of NGF, which is followed by its cleavage, and subsequent direct or indirect actions of NGF molecules triggering intricate cell signalling cascades [53,54,55]. The morphogenic effects of encapsulated NGF on hippocampal neurons observed here include accelerated neuronal development, an increased neurite outgrowth rate and neurite branching: such effects have been well established, albeit without encapsulation, in the literature pertaining to the functional properties of NGF [56,57,58,59]. The capability to dictate the direction of neurite outgrowth involves the chemoattractive action of NGF molecules on the axonal growth cone: this action relies on the concentration gradients of NGF present in the surrounding medium. This gradient can vary from a minimum of 15 ng mL^−1^ mm^−1^ (required for directed neural progenitor cells navigation [60]), with a similar level for sensory axon guidance [61], to 133 ng mL^−1^ mm^−1^ (neurite outgrowth of PC12 cells [62]). However, there are threshold concentration values (above 995 ng/mL) above which the NGF receptors become saturated, thus limiting successful neurites guidance [62]. The latter lends further support to the idea of having local, slow-releasing carriers to deliver neurotrophins, such as NGF, to brain neurons. Our data show the effects of encapsulated NGF mainly on neurons that occur at a relatively short distance (<50 µm) from the NGF-loaded microcapsules or their clusters. This suggests that the slow NGF release generates a focal concentration gradient enabling a highly localised morphogenic action. These observations underline the advantage of the site-specific directional action of neurotrophins released at a low rate from microcapsules, as opposed to some other delivery strategies, such as, for instance, triggered cargo release with external stimuli (applied focused ultrasound, light, etc. [63,64]).

Here, the release kinetics of encapsulated NGF is expected to be similar to that of the supplementing protein (i.e., BSA), the feature that has been explored in some detail in our previous studies involving encapsulation of growth factors [23,33]. The release rates of fibroblast growth factor co-encapsulated with BSA have also been investigated by an independent group [32]. Taken together, these and other studies indicate that microcapsules based on the same polyelectrolytes as here (PArg and DS) provide a slow, prolonged (up to several days) release of growth factors, with a molecular weight similar to that of NGF, over several days. In terms of its neurobiological activity, NGF shows a fairly stable effect over at least an order of magnitude of its concentration in tissue [34,35], whereas the spatial location of microcapsules in tissue must dwarf other factors in setting the local NGF concentration gradient. These considerations allow for a wide range of NGF release rates to be relevant in the context of our experiments. As shown by our recent investigation [24], empty LbL-microcapsules of the same composite had no effect on electrophysiological properties of primary hippocampal neurons even when applied intracellularly. We therefore did not use empty-capsule control in the present study.

Emerging evidence suggests that neurotrophins have also been involved in directing axonal and dendritic arborization, formation of dendritic spines and functional synaptic connections, as well as release of neurotransmitters [65,66,67], which together shapes the architecture of neural networks in the adult. We found that encapsulated NGF promoted branching of neurites in local hippocampal neurons, which subsequently established functional synapses on neighbouring cells at a comparatively early stage in vitro. The exact regime of NGF delivery could play a key role in neuronal morphogenesis because diverse structural and functional effects have been found in hippocampal neurons upon different neuropeptide administration modes, favouring neurite branching and synaptic potentiation driven by gradual and sustained concentration increases, as opposed to fast and transient delivery of neurotrophins [68]. In addition, long-term exposure to exogenous neurotrophins was reported to augment excitatory transmission in hippocampal neurons [69].

The slow release of encapsulated NGF relies on the slowly progressing biodegradation of the microcapsule shells within the physiological environment. This can be monitored and adjusted for the relatively fast efflux of encapsulated NGF, as fast as between 6 and 24 h of incubation. The fabricated microcapsules used here are composed of biodegradable components only, such as poly-L-arginine (PArg) and dextran sulphate sodium salt, which are approved by the FDA and equivalent European regulatory authorities. Being biodegradable and inert, this type of carrier therefore has a fairly strong potential for its use in humans.

Depletion of endogenous NGF in the brain leads to severe neurodegeneration and is a cause of various brain disorders often associated with cognitive dysfunction [1,2,70,71]. Therefore, delivery of exogenous NGF to damaged brain regions followed by its relatively slow, long-lasting release could, in principle, trigger an alleviating effect. One approach to encapsulate NGF has utilized a W/O/W emulsification method [15], which resulted in the formation of larger (>10 µm) capsules. The biodegradable poly (d, l-lactide-*co*-glycolide) microspheres loaded with NGF completely degraded after three months of implantation in the intact striatum [15], suggesting safe use inside the brain. A similar NGF encapsulation strategy exploiting emulsification has been advocated to promote survival of cholinergic neurons: implantation of NGF-loaded microspheres into the brain improved spatial learning and memory in the rat model of Alzheimer’s disease [72]. Nonetheless, the LbL-microcapsules seem to have certain advantages over microspheres, such as no cargo exposure to organic solvents, better tissue infiltration (due to a relatively smaller size), facilitated cargo loading, and manageable release properties.

## 5. Conclusions

The present study provides experimental evidence for the morphogenic effects of the neuropeptide NGF, encapsulated inside biodegradable LbL-microcapsules, on primary hippocampal neurons. Our findings demonstrate, firstly, the applicability of this type of carriers for localized delivery of bioactive compounds, such as neurotrophins, to the environment of nerve cells. Secondly, they suggest that the targeted delivery of NGF-loaded microcapsules has the potential to provide axonal guidance and synapse formation for central neurons during development. The key application strategy that we foresee for NGF-loaded capsules is not related to their systemic application or blood-brain barrier issues. We see the potential use of such microcapsules as a tool to help enable axonal outgrowth, trajectory guidance, and synapse formation among damaged or malformed nerve fibres *in situ*. We believe that it is by placing the capsules directly (via surgery or micro-injection) in the region(s) requiring axonal reconstruction, with high spatial precision, that the effect could be achieved. Clearly, further studies in vivo would be necessary to confirm the cargo system applicability in an intact brain.

## Data Availability

Original data are available on request from the corresponding author.

## Abbreviations

BDNF	brain-derived neurotrophic facto
BSA	bovine serum albumin
DIC	differential interference contrast microscopy
DS	dextran sulfate sodium salt
FITC	fluorescein isothiocyanate
FM1-43 dye	N-(3-Triethylammoniumpropyl)-4-(4-(Dibutylamino) Styryl) Pyridinium Dibromide
GDNF	glial-cell-derived neurotrophic factor
iPSC	induced pluripotent stem cells
KCl	high-potassium medium
LbL	layer-by-layer technique
NGF	nerve growth factor
Parg	poly-L-arginine
PLGA	poly-lactic-co-glycolic-acid
ROI	region of interest
SEM	scanning electron microscopy
s.e.m.	standard error of the mean
TRITC	tetramethylrhodamine
